# Three-Phases Interface Induced Local Alkalinity Generation Enables Electrocatalytic Glucose Oxidation in Neutral Electrolyte

**DOI:** 10.3389/fbioe.2022.909187

**Published:** 2022-04-28

**Authors:** Yangru Chen, Jun Zhang, Zhenyao Ding, Liping Chen, Haili Wang, Man Zhang, Xinjian Feng

**Affiliations:** ^1^ College of Chemistry, Chemical Engineering and Materials Science, Soochow University, Suzhou, China; ^2^ Innovation Center for Chemical Science, Soochow University, Suzhou, China

**Keywords:** hydrophobicity, three-phase interfaces, local microenvironment, electrocatalytic glucose oxidation, non-enzymatic detection

## Abstract

Electrocatalytic glucose oxidation is crucial to the development of non-enzymatic sensors, an attractive alternative for enzymatic biosensors. However, due to OH^−^ consumption during the catalytic process, non-enzymatic detection generally requires electrolytes having an alkaline pH value, limiting its practical application since biofluids are neutral. Herein, *via* interfacial microenvironment design, we addressed this limitation by developing a non-enzymatic sensor with an air–solid–liquid triphase interface electrodes that synergistically integrates the functions of local alkalinity generation and electrocatalytic glucose oxidation. A sufficiently high local pH value was achieved *via* oxygen reduction reaction at the triphase interface, which consequently enabled the electrochemical oxidation (detection) of glucose in neutral solution. Moreover, we found that the linear detection range and sensitivity of triphase non-enzymatic sensor can be tuned by changing the electrocatalysts of the detection electrode. The triphase electrode architecture provides a new platform for further exploration and promotes practical application of non-enzymatic sensors.

## Introduction

Diabetes is a chronic disease that threatens human health across the world. Notably, over 420 million adults worldwide diabetic (Rubino, 2016; Lu et al., 2016; Ohayon et al., 2020; Zhou Y et al., 2020; Zhang et al., 2021). Reliable glucose monitoring facilitates better blood glucose control and prevents complications. Enzymatic electrochemical biosensors have been widely used for glucose detection; however, biological enzymes are susceptible to factors, such as temperature, pH, and ions, hindering the stability and scope of enzymatic biosensors (Yang et al., 2014; Sun and James, 2015; Johnston et al., 2021). Non-enzymatic sensor based on direct electrocatalytic glucose oxidation reaction, is highly attractive as it avoids the use of biological enzymes (Zhang et al., 2018; Teymourian et al., 2020). In the past decades, great efforts have been devoted to the development of electrocatalysts, and a variety of electrocatalytic materials including noble metal (Lang et al., 2013; Bae et al., 2019), metal alloys (Yamauchi et al., 2012; Bag et al., 2020), metal oxides (Cheng et al., 2016; Mondal et al., 2017) and carbon-related materials (Bao et al., 2017; Dung et al., 2013) have been reported. Unfortunately, due to OH^−^ consumption during the electrochemical glucose oxidation process, C_6_H_12_O_6_ (glucose) + 2OH^−^→ C_6_H_10_O_6_ (glucolactone) + 2H_2_O+ 2e^−^, non-enzymatic sensors generally require solutions with high pH value for acceptable sensitivity and linear detection range (Wei et al., 2020). In addition, serious electrode fouling will occur during the oxidation reaction, due to the lack of sufficient OH^−^ supply (Adeel et al., 2021; Chen et al., 2019). With these restrictions, the development and practical application of non-enzymatic sensors has been limited since biofluids are neutral.

**FIGURE 1:**
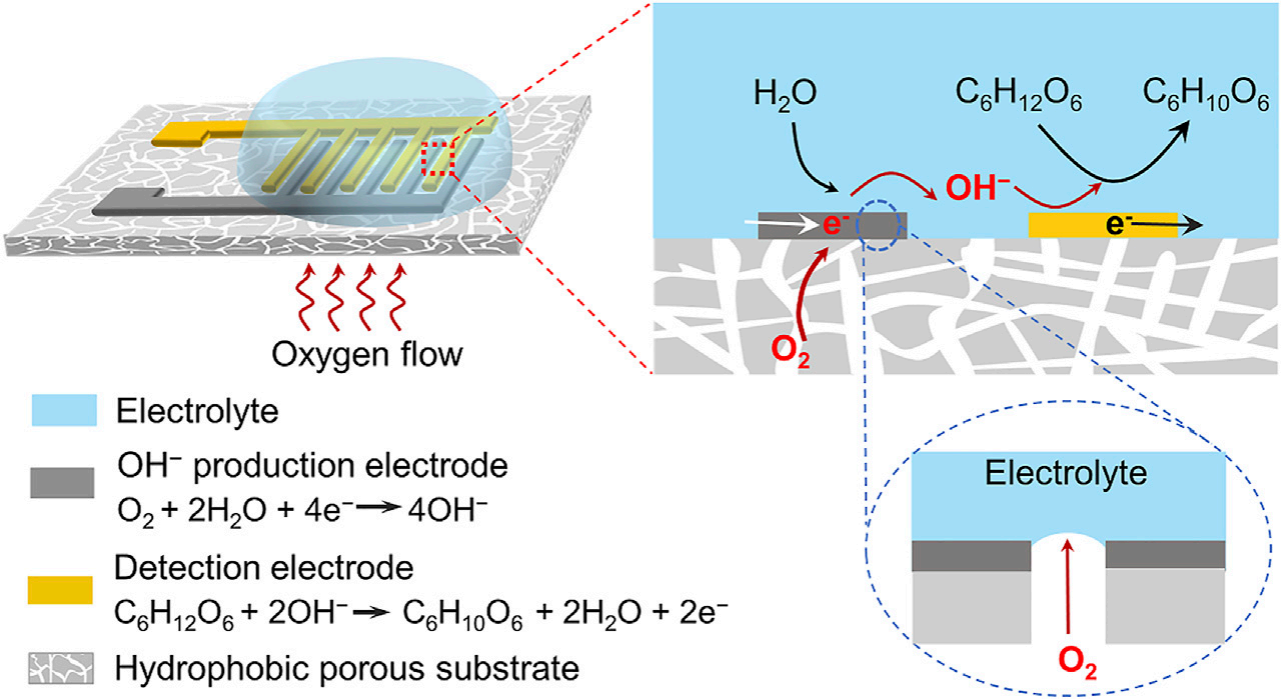
Schematic illustration of the triphase non-enzymatic sensor. The sensor consists of a hydrophobic porous substrate, an electrode for local alkalinity generation *via* oxygen reduction reaction, and an electrode for electrocatalytic glucose oxidation reaction. Sufficient oxygen supplied from the air phase was reduced to OH^−^ at the triphase interface, leading to a high interface pH for electrocatalytic glucose oxidation. The electrode architecture makes the non-enzymatic glucose detection independent of the solution pH. During the experiment, a negative potential was first applied to the OH^−^ production electrode to generate a local alkaline microenvironment, and then a positive potential was applied to the detection electrode for electrochemical glucose oxidation.

Besides the electrocatalytic materials, the reaction interface microenvironment that governs the diffusion, adsorption and reaction of reactants is also crucial to the performance of catalytic reaction, but has received limited attentions (Sheng et al., 2017; Song et al., 2018; Zhou H et al., 2020; Kim et al., 2021; Yang and Gao, 2022). In this work, we addressed this limitation by developing a novel non-enzymatic sensing system with an air–solid–liquid triphase interface as illustrated in Figure 1. This electrode architecture synergistically integrates the functions of interfacial local alkalinity generation and electrocatalytic glucose oxidation. Two electrodes that were used for local OH^−^ production and glucose detection, respectively, and they were deposited on a hydrophobic porous substrate in an interdigitated shape. When the sensing system was immersed in an aqueous solution, water contacted the electrode surface but did not enter the inner porous substrate, due to its surface hydrophobicity (Wen et al., 2015; Liu et al., 2017). This led to the formation of an air–solid–liquid triphase interface where sufficient oxygen can be supplied directly from the air phase. Oxygen can be readily reduced to OH^−^ at the surface of electrocatalysts, O_2_+ 2H_2_O+ 4e^−^→ 4OH^−^, leading to an increase in the local pH. Using the triphase electrode architecture, sufficient oxygen was utilized to generate OH^−^ and form a sufficient high local alkaline microenvironment, enabling the electrocatalytic glucose oxidation in neutral solution.

## Materials and Methods

### Materials

The hydrophobic porous polyethylene (PE) membrane was purchased from Entek International LLC and the hydrophilic flat (pore-free) polyethylene terephthalate (PET) membrane was purchased from Membrane solutions. Sodium sulfate, sodium hydroxide, sulfuric acid, phenolphthalein, chloroauric acid, sodium chloride, lactic acid, galactose, glucose, ascorbic acid and acetaminophen were purchased from Sinopharm Chemical Reagent. All reagents are analytical grade. Nafion perfluorinated resin solution (5 wt% in lower aliphatic alcohols and water, contained 15–20% water) was purchased from Sigma-Aldrich. The high purity platinum target material (99.95%) was purchased from Shijiazhuang Dongming New Material Technology Co., Ltd. All of our experiments used deionized water. All reagents are used as received reagents without further purification.

### Fabrication of Triphase/Diphase Electrode

1) The hydrophobic porous PE membrane was cut into a rectangle, cleaned with alcohol 3–4 times and dried with Ar, then was tightly against an interdigital electrode mask and directly deposited by automatic sputter coater (GVC-2000, Hezao) of a platinum target for 300 s at 30 mA. Thus, a triphase Pt-Pt electrode was prepared. (2) Au electrocatalysts were electrodeposited onto the Pt detection electrode at 0 V *vs*. Ag/AgCl for 100, 200, 400 and 600 s in 5 mM chloroauric acid solution (10 g/L in DI water), respectively. Then, a triphase Pt-Au electrode was prepared. (3) The 50 μL mixed solution of Nafion (5 wt% in DI water) drop cast onto the triphase Pt-Au electrode with an area of 0.7 cm × 1.0 cm and dried in an oven for 0.5 h at 60°C. For controlled experiment, a diphase Pt-Pt electrode was also prepared in a similar way using a hydrophilic non-porous PET membrane as substrate.

### Characterization

The morphology was characterized by FE-SEM (SU8010, Hitachi) and the element mapping distribution is characterized by Evo-SEM (EVO18, Zeiss). The water contact angle was measured by a contact angle goniometer (Jc 2000d6, Poareach). Electrochemical measurements were carried out at room temperature using the CHI 660E workstation (CH Instruments, Inc.).

### Measurement Methods

Electrochemical measurements were performed using a CHI 660E electrochemical workstation with a three-electrode system. The triphase/diphase electrode consisting of an OH^−^ production electrode and a glucose detection electrode was used as the working electrode. A Pt wire was as the counter electrode and an Ag/AgCl (3 M KCl) was as the reference electrode. Na_2_SO_4_ solution was used as the electrolyte. 1) The potential of OH^−^ production was determined by linear sweep voltammetry in Ar or O_2_ atmosphere, at a scan rate of 50 mV s^−1^. 2) The pH-potential curve measurement was conducted using chronopotentiometer with a current of 5 μA for 30 s in solutions with different pH. The dynamic surface pH of the detection electrode was carried out using chronopotentiometer as mentioned above after the OH^−^ production step. 3) Two steps to the working electrode were used to measure glucose concentrations, including a negative potential of −0.6 V *vs*. Ag/AgCl for 20 s on the OH^−^ production electrode and 0.4 V *vs*. Ag/AgCl for 10 s on the detection electrode. 4) Selectivity tests were performed by amperometric measurement at 0.4 V after OH^−^ production step. A series of interferents (50 μM of ascorbic acid, lactic acid, galactose, acetaminophen and sodium chloride) were added to the solution after the addition of 0.5 mM glucose using the triphase Nafion-coated Pt-Au electrode.

## Results and Discussion

**FIGURE 2:**
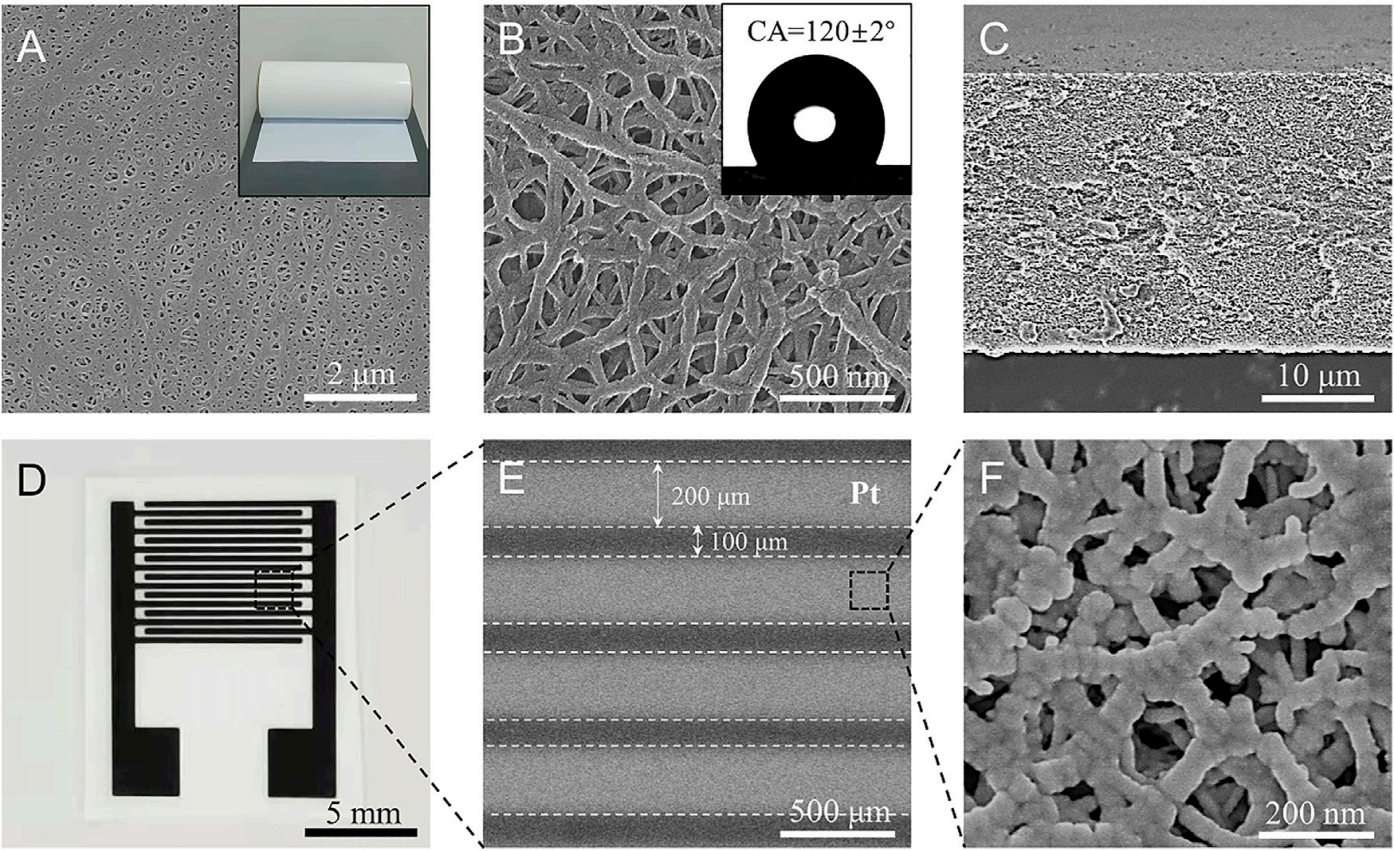
**(A,B)** Scanning electron microscopy (SEM) images of the porous polyethylene membrane substrate at low and high magnification, respectively. Insets in **(A,B)** show photographs of the membrane and water droplets on it with a contact angle of about 120 ± 2°. **(C)** Cross-section SEM image of the membrane with a thickness of about 25 μm. **(D)** Photograph of Pt-Pt electrodes sputtered on the porous substrate with eight pairs of electrodes. **(E,F)** are SEM images of the Pt electrode at low and higher magnifications. The electrode band-width is 200 μm, the gap between the interdigitated electrodes is 100 μm. The Pt electrode also has a porous structure.

The triphase electrode illustrated in Figure 1 was constructed by choosing a hydrophobic porous polyethylene (PE) membrane (Figure 2A) as the substrate. The PE membrane has an average pore size of about 200 nm (Figure 2B) and a thickness of about 25 μm (Figure 2C). Contact angle (CA) analysis of the PE membrane shows a CA of about 120 ± 2° (inset of Figure 2B), indicating a hydrophobic surface property. Platinum (Pt) metal, with good oxygen reduction and electrochemical glucose oxidation capabilities (Briega-Martos et al., 2017; Lee et al., 2018), was chosen as a model electrocatalyst to prepare the Pt-Pt electrode. As shown in Figure 2D,E and Supplementary Figure S1, the Pt-Pt electrode has eight pairs of electrodes, a width of 200 μm and a gap distance of 100 μm. After Pt deposition the porous structure of the PE substrate was maintained (Figure 2F), which facilitates rapid oxygen transport from the free space of the porous hydrophobic PE membrane to the electrode surface. For the control experiment, a conventional solid–liquid diphase electrode was also fabricated on a non-porous flat hydrophilic polyethylene terephthalate (PET) substrate (Supplementary Figure S2).

**FIGURE 3:**
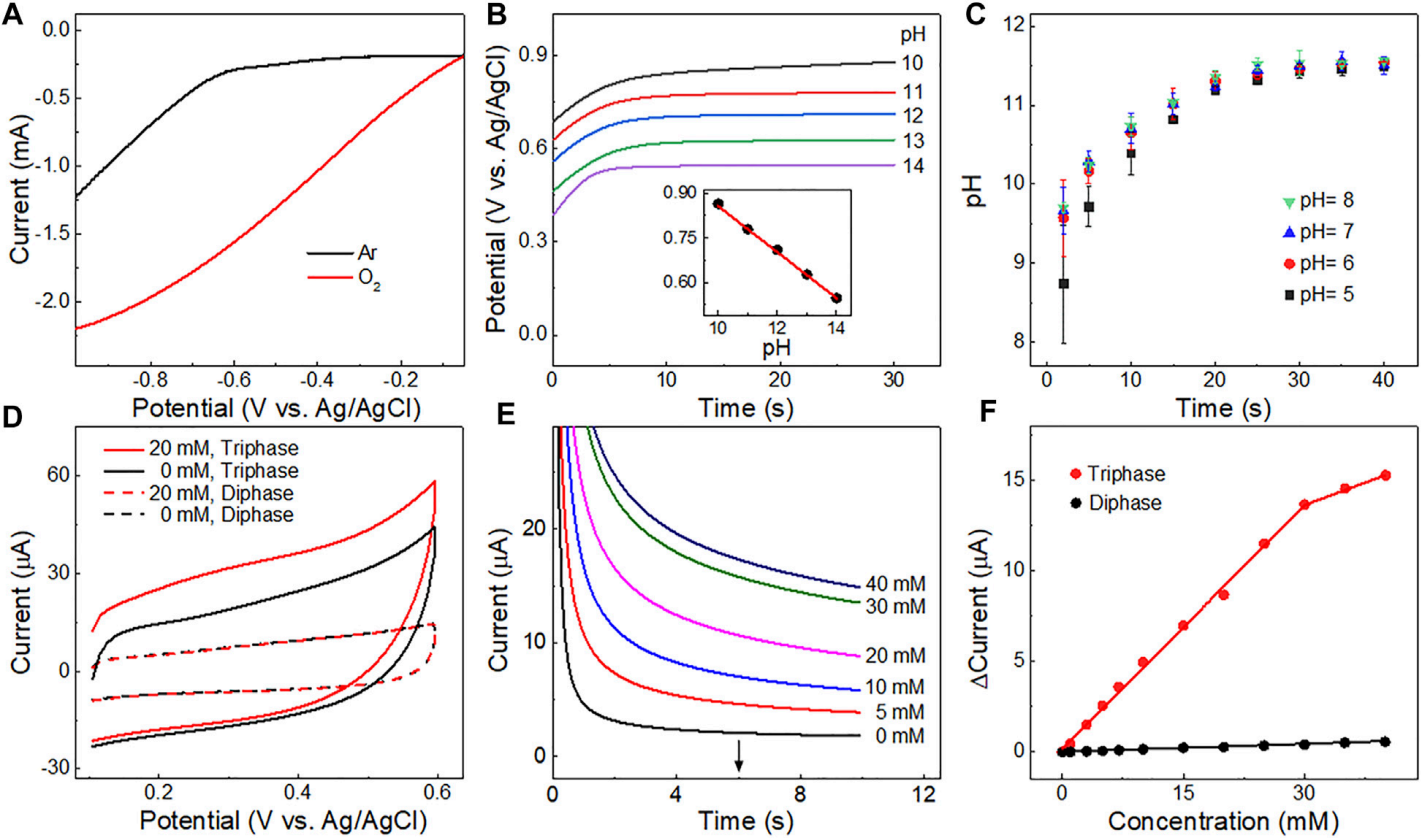
**(A)** Linear sweep voltammetry curves of the Pt detection electrode in Ar and O_2_ saturated 0.10 M Na_2_SO_4_ solution. **(B)** Potentials of the detection electrode measured in solutions of different pH with a constant current (5 μA). Inset is the linear relationship between potentials derived at 20 s and solution pH values. **(C)** Changes in pH value over time at the detection electrode upon application of a potential of −0.6 V at the OH^−^ production electrode in electrolyte with different pH values. The error bar represents the standard deviation for two replicated measurements. **(D)** Cyclic voltammetry curves obtained in a 0.1 M Na_2_SO_4_ solution with or without 20 mM glucose using a triphase or diphase Pt-Pt electrode after the OH^−^ production step. **(E)** Amperometric i-t curves corresponding to the triphase Pt-Pt electrode with glucose concentrations up to 40 mM. **(F)** Corresponding calibration plots of triphase and diphase electrodes derived from **(E)** and Supplementary Figure S5 at 6 s ∆Current = Current_s_ - Current_0_; Current_0_ is the background current; and Current_s_ is the current measured in the solution with different glucose concentrations.

The performance of the triphase electrode for local alkalinity generation was first investigated. Figure 3A shows linear sweep voltammetry (LSV) of Pt electrode in 0.1 M sodium sulfate (Na_2_SO_4_) solution saturated with Ar or O_2_. Water reduction was apparent when the potential was negative than approximately −0.6 V *vs.* Ag/AgCl (black cure in Figure 3A), while a much higher potential was sufficient for O_2_ reduction to OH^−^ (red curve in Figure 3A; Supplementary Figure S3). In order to obtain the high OH^−^ production capacity and avoid the hydrogen evolution reaction to generate hydrogen bubbles, which would affect glucose transmission and the accuracy of detection, a potential of −0.6 V (*vs*. Ag/AgCl) was chosen for OH^−^ production in this work. The OH^−^ ions produced diffused outwards and led to an increase in pH near the surface of the adjacent glucose detection electrode.

To determine the pH value at the surface of the detection electrode after OH^−^ production, a chronopotentiometer was used to apply a constant current (5 μA) at the detection electrode in solutions with different pH values (10.0–14.0) (Figure 3B). The relationship between the pH values and the potentials was recorded (inset of Figure 3B) according to the effect of OH^−^ concentration on the potential of the oxygen evolution reaction (4OH^−^→ 2H_2_O + O_2_+ 4e^−^) (Dresp et al., 2021; Maruthapandian et al., 2017; Yin and Liu, 2020). Thus, the dynamic pH value near the surface of the Pt detection electrode was measured after OH^−^ production. As shown in Figure 3C, the measured interfacial pH values were all above 11 for solutions with bulk pH of 5–8.0 when the time course of OH^−^ production step is longer than 20 s. Thus, to ensure the reproducibility for further experiments a time course of 20 s was chosen for the OH^−^ production step.

The triphase Pt-Pt electrode was then used for glucose detection (Supplementary Figure S4). The cyclic voltammograms of the triphase or diphase detection electrodes in 0.10 M Na_2_SO_4_, with or without 20 mM glucose after OH^−^ production step, are shown in Figure 3D. The anodic current increased upon the addition of glucose for the triphase electrode (red solid curve), while a negligible anodic current increase was recorded (red dotted curve) for the diphase electrode, due to the lack of sufficient oxygen and the insufficient alkalinity needed for electrocatalytic glucose oxidation. Figure 3E shows that the anodic current response of the triphase electrode increased with glucose concentration to approximately 40 mM. The current response versus glucose concentration (Figure 3F) shows that the triphase electrode displayed a linear detection range to about 30 mM (red line), and the sensitivity was 2.1 μA mM^−1^ cm^−2^. In remarkable contrast, negligible current increase was observed for the diphase electrode as the glucose concentration increased (Figure 3F; Supplementary Figure S5). These results confirm sufficient oxygen was supplied at the triphase interface, and non-enzymatic glucose detection was achieved in neutral solution.

**FIGURE 4:**
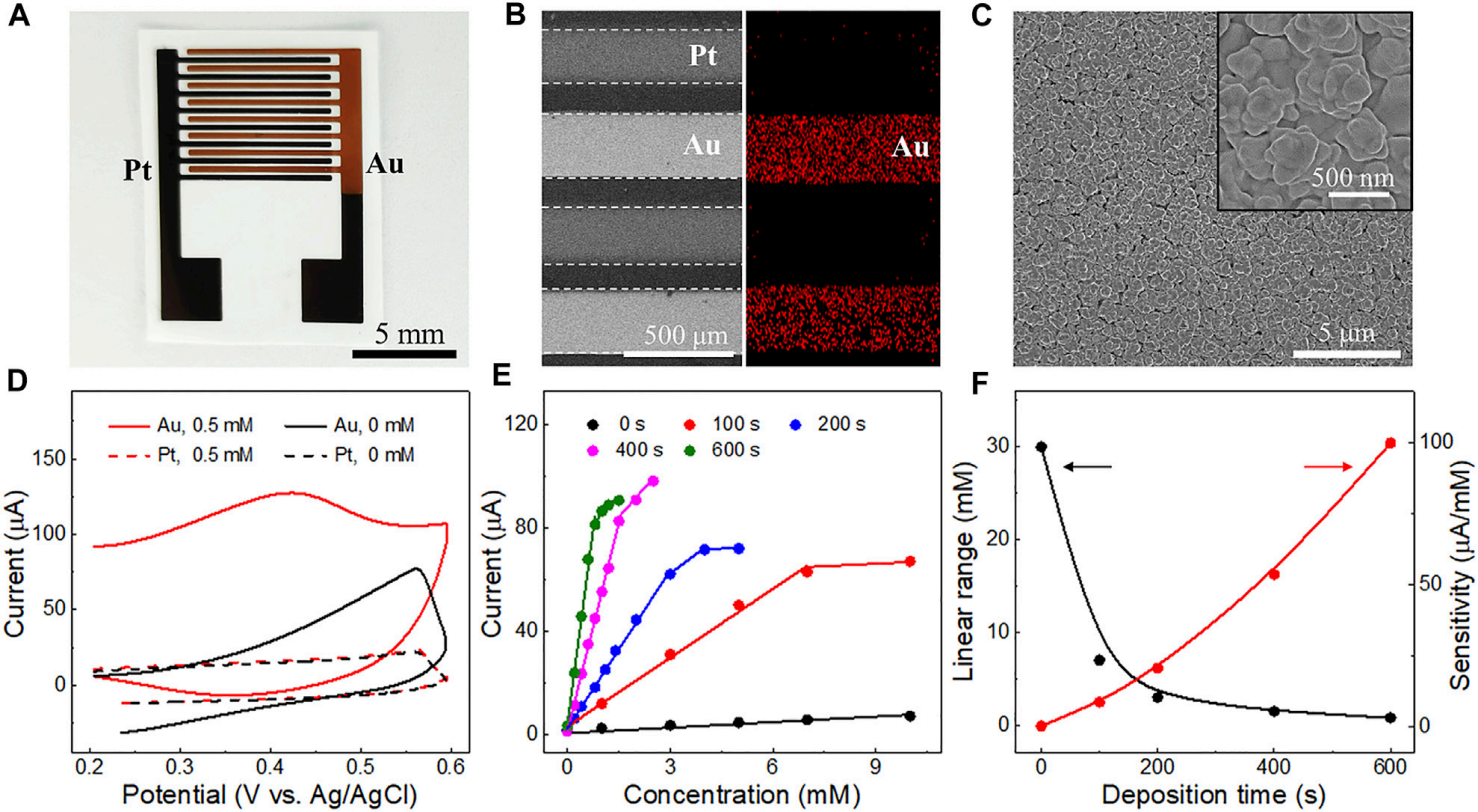
**(A)** Photograph of the triphase electrodes after 400 s Au electrocatalyst deposition on the Pt detection electrode. **(B)** SEM image of the triphase Pt-Au electrodes (left) and the corresponding Au elemental mapping distribution (right). **(C)** SEM images of the Au nanoparticles with diameter ranging from 200 to 500 nm. The inset is an enlarged SEM view of the Au nanoparticles. **(D)** Cyclic voltammetry curves of the Pt-Pt and Pt-Au electrodes obtained in solutions with or without glucose (0.5 mM). **(E)** Corresponding calibration plots of the electrodes with different Au electrodeposition times (100, 200, 400 and 600 s) derived from their amperometric i-t curves (Supplementary Figure S7) at 6 s. **(F)** Relationship between the linear detection range (black curve) and the sensitivity (red curve) of electrodes with different Au electrodeposition times.

The performance of the triphase non-enzymatic sensor can be further improved by alternating the electrocatalysts of the detection electrode. Au is a commonly used and stable electrocatalyst with high activity towards glucose oxidation in alkaline solution (Zhong et al., 2017). To improve the device performance, different amounts of Au nanoparticles were electrodeposited on the detection electrode (Pt) by adjusting deposition time. The morphologies of the Pt-Au electrode were further characterized. As shown in Figure 4A, the color of the detection electrode turned from black to dark yellow after Au catalyst deposition. Figure 4B shows SEM image of the Pt-Au electrode. The existence and distribution of the Au catalysts were confirmed by elemental mapping (Figure 4B, right) and energy dispersive X-ray spectroscopy (EDS) analysis (Supplementary Figure S6). Au particles have a diameter of 200-500 nm (Figure 4C) and were uniformly distributed on the surface of the detection electrode.

We then explored the performance of the triphase electrode after Au deposition. As shown in Figure 4D, a strong anode wave (red solid curve) is observed on the detection electrode at 0.4 V (*vs*. Ag/AgCl), due to the carbonyl oxidation of glucose. Interestingly, its glucose oxidation current was much higher than that of the detection Pt electrode without Au electrocatalyst (red dotted curve). This indicates that the introduction of Au can effectively improve the performance of glucose oxidation. Thus, the performance of Pt-Au electrodes with different Au electrodeposition times (100, 200, 400 and 600 s) was further investigated. As shown in Figure 4E and Supplementary Figure S7, with the increase in electrodeposition time, the sensitivity of the electrode for glucose detection increased, but the linear detection upper limit decreased (Figure 4F). We reasoned that increasing the amount of Au catalysts would increase the number of available active sites for glucose oxidation and consequently lead to a higher reaction rate and sensitivity. A higher reaction rate generally results in faster OH^−^ consumption at the interface microenvironment and makes the detection of glucose at high concentrations difficult. These results indicate that glucose detection with different sensitivity and linear range can be obtained by modifying the electrocatalysts on the detection electrode.

**FIGURE 5:**
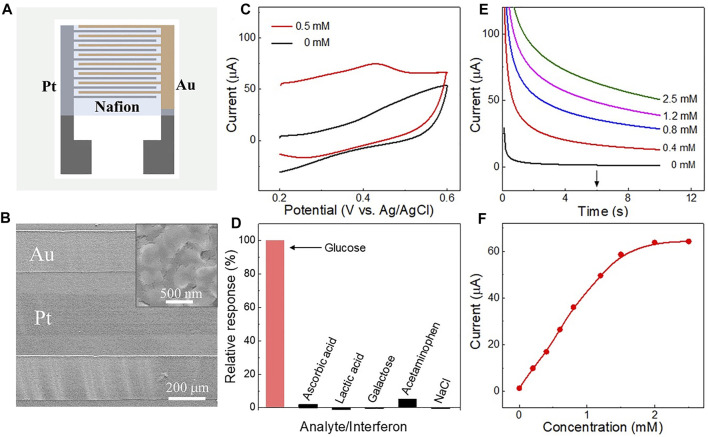
**(A)** Schematic of the Nafion layer on the Pt-Au electrode surface. **(B)** SEM image of the Nafion film-coated electrode. The inset is an enlarged view of the surface of the Au electrode. **(C)** Cyclic voltammetry curves obtained in a 0.1 M Na_2_SO_4_ solution with or without 0.5 mM glucose, using the nafion-coated triphase Pt-Au electrode, respectively. **(D)** Histogram of the interference effects on the electrode while measuring 0.5 mM glucose at 0.4 V *vs*. Ag/AgCl. The concentration of interferents was 0.05 mM, including ascorbic acid, lactic acid, galactose, acetaminophen and sodium chloride (NaCl). **(E)** Amperometric i-t curves corresponding to the nafion-coated electrode with glucose concentrations up to 2.5 mM. **(F)** Corresponding calibration plots of nafion-coated triphase electrode derived from **(E)** at 6 s.

Apart from the linear dynamic range and sensitivity, selectivity is also a crucial parameter for non-enzymatic sensors. To reduce the influence of interferents, the surface of the Pt-Au electrode was coated with a layer of Nafion, as illustrated in Figure 5A. Figure 5B is a SEM top view of the Nafion film on the Pt-Au electrode surface. The negatively charged Nafion film can selectively restrict the diffusion of some kinds of anions from the solution to the electrode surface without affecting the detection of glucose (Cao et al., 2013; Chen et al., 2015). The performance of nafion-coated Pt-Au electrode was evaluated after the OH^−^ production step. As shown in Figure 5C, the glucose oxidation current of the nafion-coated triphase Pt-Au electrode significantly increased after 0.5 mM glucose addition (red curve).

Interfering compounds, including ascorbic acid, lactic acid, galactose, acetaminophen, and sodium chloride (NaCl), were then added into the sample matrix in large excess compared with that in human perspiration (Lu et al., 2019; Zhu et al., 2019). As shown in Figure 5D, negligible interferences were observed with the triphase Pt-Au electrode while measuring glucose in the presence of interfering compounds. Figure 5E shows the electrode responses in glucose solution with concentrations up to 2.5 mM. A linear detection upper limit of about 1.5 mM and sensitivity of 179.1 μA mM^−1^ cm^−2^ were obtained (Figure 5F). This result indicates that the Nafion layer on the electrode surface can not only reduce the effects of some kinds of interference but also ensure the detection of glucose. In addition, the repeatability of the nafion-coated electrode was also assessed. Supplementary Figure S8 shows 100 successive measurements of 0.5 mM glucose using the same biosensor. A relative standard deviation of only 2.34% was observed for these measurements, indicating good repeatability.

## Conclusion

In summary, we have fabricated a triphase electrode that enables electrocatalytic glucose oxidation and non-enzymatic sensing in neutral solution. Using the air-solid-liquid triphase electrode, sufficient oxygen was available from the air phase for the generation of a local interfacial alkaline microenvironment *via* oxygen reduction reaction. This sensor is superior to other non-enzymatic ones because it does not require an electrolyte with high pH. Moreover, the triphase non-enzymatic electrode with tunable performance including sensitivity and linearity, can be obtained by choosing suitable electrocatalysts, which endows great potential for practical applications in different scenarios.

## Data Availability

The original contributions presented in the study are included in the article/Supplementary Material, further inquiries can be directed to the corresponding authors.
