# Supplementary material for: Three-Phases Interface Induced Local Alkalinity Generation Enables Electrocatalytic Glucose Oxidation in Neutral Electrolyte
Source: Front Bioeng Biotechnol. 2022 Apr 28;10:909187. doi: 10.3389/fbioe.2022.909187 (PMC9096097; doi:10.3389/fbioe.2022.909187)
Supplement: Supplementary file 1 [file DataSheet1.pdf]

Three-phases Interface Induced Local Alkalinity Generation Enables Electrocatalytic Glucose Oxidation in Neutral Electrolyte

Yangru Chen[1], Jun Zhang[1]*, Zhenyao Ding[1], Liping Chen[1], Haili Wang[1], Man Zhang[1], Xinjian Feng[1,2]*

[1]College of Chemistry, Chemical Engineering and Materials Science, Soochow University, Suzhou 215123, P. R. China

[2] Innovation Center for Chemical Science, Soochow University, Suzhou, 215123, Jiangsu Province, China

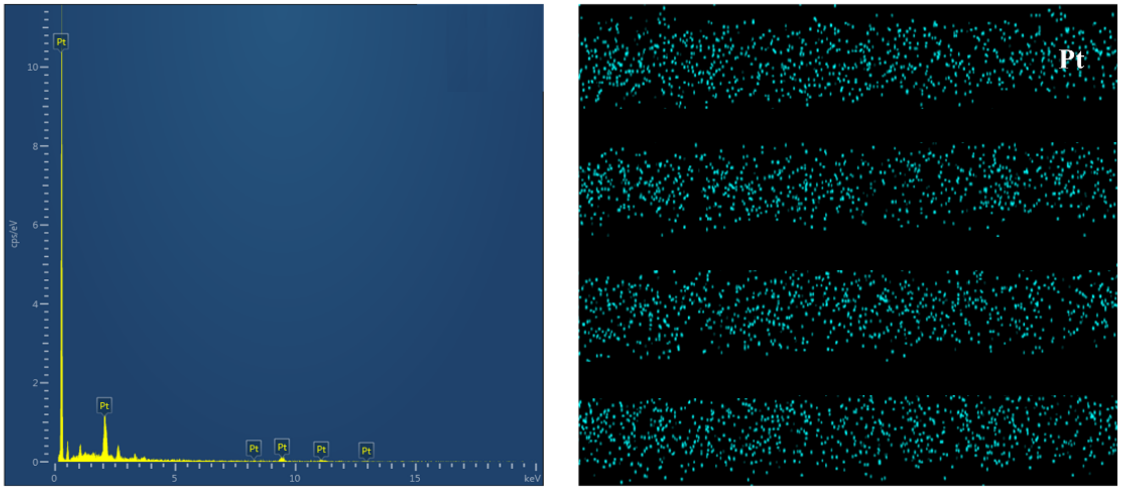

Figure S1. The energy dispersive X-ray spectroscopy (EDS) linear scan curve (left) and elemental mapping distribution (right) of the triphase Pt-Pt electrode.

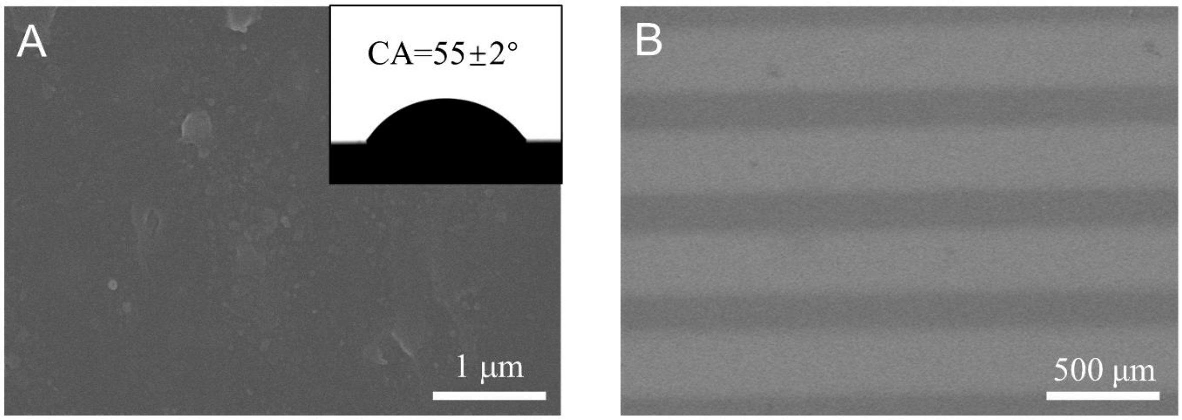

Figure S2. (A) Scanning electron microscopy (SEM) top view of a hydrophilic PET substrate. The inset shows a water droplet placed on the substrate with a CA of about 55 ± 2°. **(B)** SEM images of the diphase Pt-Pt electrode. The inset is an enlarged view of the Pt particles.

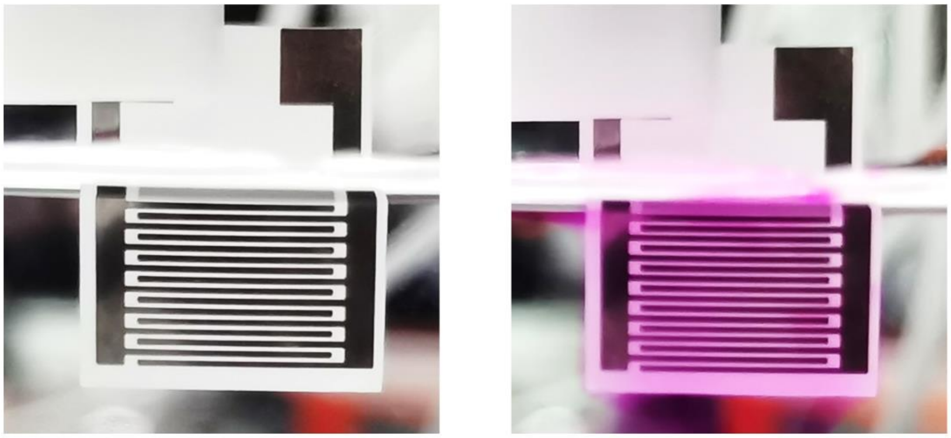

Figure S3. Photographs for the phenolphthalein indicator test of the triphase Pt-Pt electrode. The substrate was dipped in solution containing 0.1 M Na_2SO_4 and phenolphthalein indicator. The pH test experiment was carried out at −0.6 V vs Ag/AgCl on the OH^--production electrode for 30 s, which intuitively proves OH^- generation through color change.

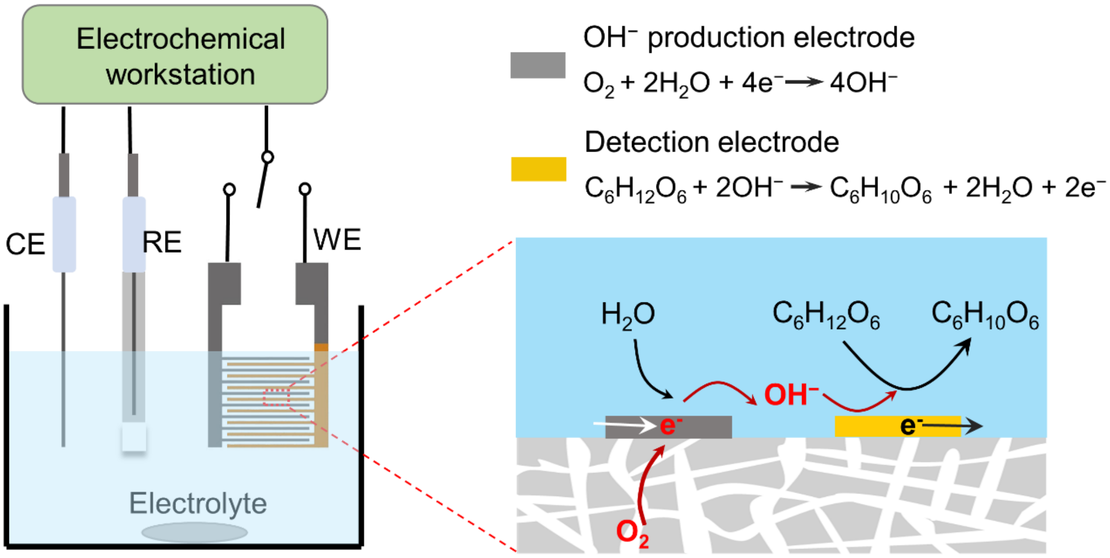

Figure S4. Schematic of an electrochemical test of the triphase non-enzymatic sensor.

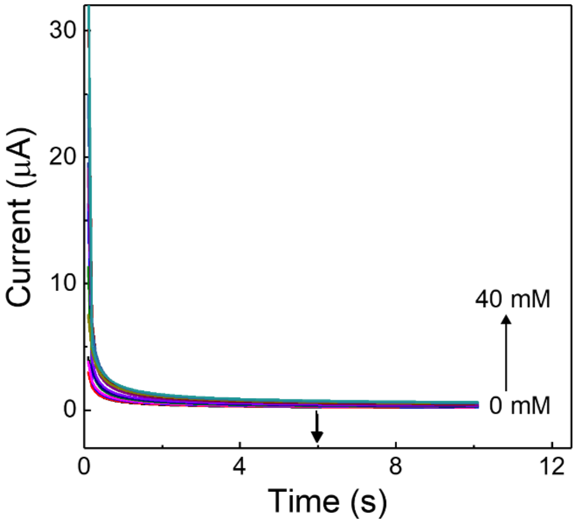

Figure S5. Amperometric i-t curves corresponding to the diphase interdigitated electrode with glucose concentrations up to 40 mM.

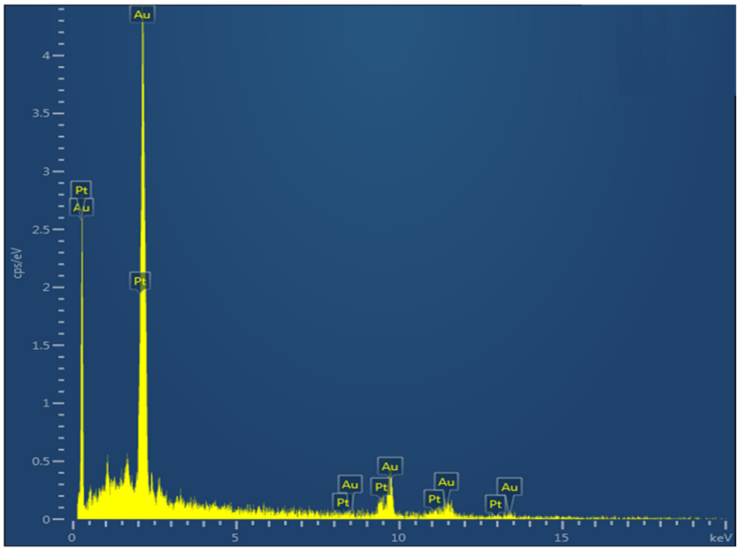

Figure S6. EDS linear scan curve of the triphase Pt-Au electrode, showing both platinum and gold peaks.

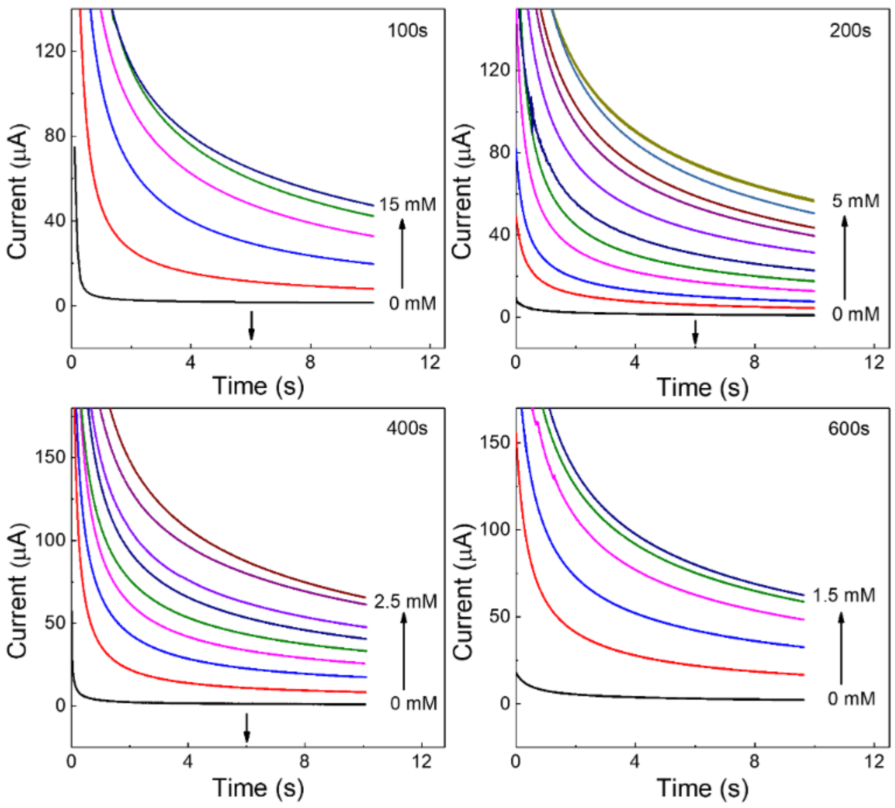

Figure S7. Amperometric i-t curves corresponding to the glucose-detection electrodes with 100, 200, 400 and 600 s Au electrodeposition time.

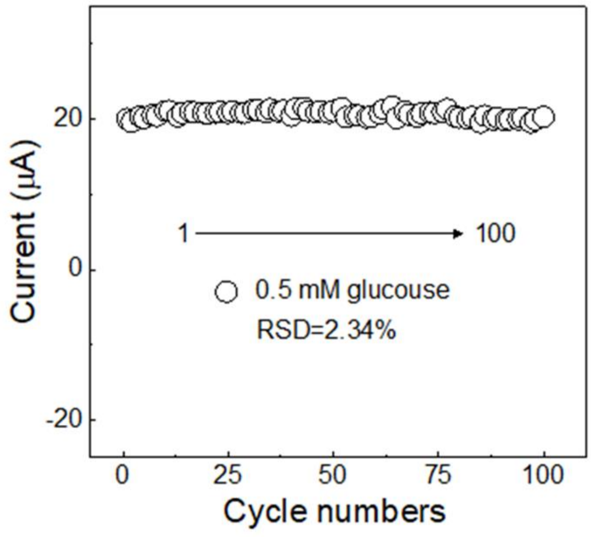

Figure S8. The nafion-coated triphase Pt-Au electrode measured 100 times in 0.5 mM glucose. The relative standard deviation is 2.34%.
